# Assessment of Factors Related to Sarcopenia in Patients with Systemic Sclerosis

**DOI:** 10.3390/jcm14051573

**Published:** 2025-02-26

**Authors:** Tuba Yuce Inel, Gozde Dervis Hakim, Merih Birlik

**Affiliations:** 1 Division of Rheumatology, Izmir City Hospital, Izmir 35530, Turkey; 2 Division of Gastroenterology, Izmir City Hospital, Izmir 35530, Turkey; gozde.dervis@deu.edu.tr; 3 Division of Rheumatology, Dokuz Eylul University, Izmir 35360, Turkey; birlikm@deu.edu.tr

**Keywords:** systemic sclerosis, sarcopenia, older patients, SARC-F, fat-free mass index

## Abstract

**Objectives**: Systemic sclerosis (SSc) patients exhibit a heightened vulnerability to sarcopenia, a condition characterized by the loss of muscle mass and strength. This study aims to determine the prevalence of sarcopenia in patients with SSc and to investigate the associated factors contributing to this condition. **Methods**: Eighty patients with SSc were included in the study, and their demographic and clinical characteristics, body composition by bioelectrical impedance analysis, SARC-F score, chair-stand test performance, and 4 m walking speed were recorded. **Results**: Among the 80 participants, 91.3% were female, with a median age of 56.5 years (range 45–65). The majority (70%) had limited SSc, and 71.3% reported at least one comorbidity. According to the International Physical Activity Questionnaire, only 12.5% of participants met the criteria for an active lifestyle. The SARC-F questionnaire indicated that 20% of patients were at risk for sarcopenia. The prevalence of sarcopenia among patients showed considerable variability: 5% (95% CI 0.1–9) were identified through the appendicular skeletal muscle index (ASMI), 8.8% (95% CI 2.4–15) via the fat-free mass index (FFMI), and a concerning 20% (95% CI 11–29) according to the skeletal muscle mass index (SSMI). A multivariate logistic regression analysis identified age as the only factor significantly influencing the SARC-F score, with an odds ratio of 1.081 (95% CI 1.012–1.154, *p* = 0.020). Additionally, the older age group demonstrated a lower level of physical activity, poorer chair-stand test outcome, and slower 4 m gait speeds (*p* = 0.013, *p* = 0.008, *p* = 0.001, respectively), as well as a higher reported frequency of falls (*p* = 0.039). **Conclusions**: Sarcopenia is a prevalent issue among individuals with SSc, particularly in the older population. This study did not identify a direct correlation between sarcopenia and SSc subtype, disease activity, or other clinical parameters. However, the need for an improved cut-off value for diagnosing sarcopenia in this specific cohort is evident.

## 1. Introduction

**Figure 1:**
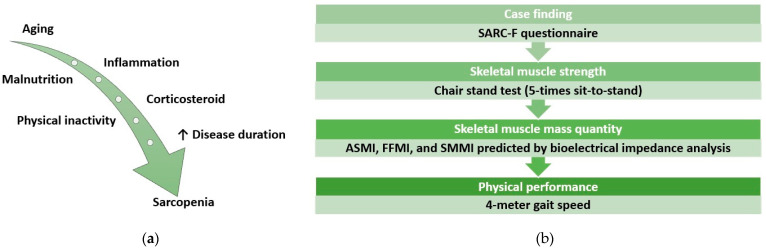
(**a**) outlines the various factors that may contribute to the development of sarcopenia, and (**b**) presents the algorithm utilized to diagnose sarcopenia.

Systemic sclerosis (SSc) is a chronic autoimmune connective tissue disease characterized by inflammation, microvascular damage, and progressive fibrosis of the skin and various internal organs, including the lungs, gastrointestinal tract, and cardiovascular system [1]. Patients with SSc are at increased risk for abnormalities in body composition due to factors such as gastrointestinal involvement, chronic inflammation, malnutrition, comorbidities, polypharmacy, and corticosteroid therapy (Figure 1a) [2]. Additionally, the involvement of the heart, lungs, and joints in individuals with SSc can lead to diminished exercise capacity and reduced physical activity [3]. These conditions are closely related to sarcopenia, which refers to the loss of muscle mass, strength, and function [4]. The prevalence of sarcopenia among SSc patients has been reported at 22.5% [2]. Sarcopenia increases the risk of falls, functional decline, and frailty, impacting quality of life and incurring higher healthcare costs [5]. The clinical predictors of sarcopenia include a prolonged disease duration, the modified Rodnan skin score (mRSS), and involvement of the esophagus [6]. A meta-analysis with subgroup analyses based on age revealed that the prevalence of sarcopenia was lower in SSc patients under 60 years (20%) compared to those over 60 years (24%) [7]. Additionally, a study focusing on relatively younger SSc patients found a prevalence of sarcopenia at 10.7% [8].

This study aimed to investigate the prevalence of sarcopenia in patients with SSc and to examine the associated factors. We collected data on muscle mass, strength, and functional performance to explore the relationship between sarcopenia and clinical characteristics of SSc, ultimately seeking to enhance the understanding of sarcopenia in this specific patient group.

## 2. Method

The study was designed as a cross-sectional investigation among adult patients with SSc who attended the rheumatology outpatient clinic. All patients met the 2013 ACR/EULAR criteria for the diagnosis of SSc [9]. Demographic data, clinical assessments, and autoantibody status were recorded. Skin involvement was evaluated using a modified Rodnan skin score [10]. Limited cutaneous (lcSSC) and diffuse cutaneous (dcSSC) systemic sclerosis were diagnosed according to the LeRoy classification [11]. The SSc patients’ disease severity and prognosis were conducted using the Medsger Systemic Sclerosis Severity Scale [12]. Patients with hypothyroidism, neurological disorders, infectious diseases, oncological conditions, severe joint contractures, and active arthritis, as well as those who are pregnant or lactating, were excluded from this study. Additionally, none of the participants had been treated with corticosteroid therapy at an equivalent dose of prednisone of 5 mg/day or higher for a minimum duration of six months.

Body mass index (BMI) was calculated from weight (kg) and height (m) and expressed in kg/m^2^. Fat-free mass (FFM) was evaluated using bioimpedance analysis (BIA), with skeletal muscle mass (SMM) calculated as follows: SMM (kg) = 0.566 × FFM [13]. Skeletal muscle mass index (SMMI) was adjusted for weight. The cut-off thresholds of SMMI were accepted as 37.4% and 33.6% in males and females [14]. The appendicular skeletal muscle mass index (ASMI) was determined by dividing the appendicular SMM (the total muscle mass in both arms and legs) by the square of height. The European Working Group on Sarcopenia in Older People (EWGSOP2) classified low muscle quantity as an ASMI below 7.0 kg/m^2^ for men and below 5.5 kg/m^2^ for women [4]. The sex-specific cut-off values for the fat-free mass index (FFMI) indicating reduced muscularity were defined as less than 15 kg/m^2^ for females and less than 17 kg/m^2^ for males [15].

The SARC-F is a 5-item questionnaire designed for patients to self-report their risk of sarcopenia [16]. It assesses symptoms related to limitations in strength, mobility, the ability to rise from a chair, climbing stairs, and instances of falls. Scores range from 0 to 10, with a score of 4 or higher identified as predictive of sarcopenia and associated with poor outcomes.

The International Physical Activity Questionnaire (IPAQ) short form was used to categorize the physical activity status of patients [17]. The 4 m gait speed [18] and chair stand test [19] assessed physical performance. According to the EWGSOP2, a gait speed cut-off of ≤0.8 m/s is recommended to indicate severe sarcopenia [4]. The diagnostic algorithm utilized for detecting sarcopenia is illustrated in Figure 1b, and evaluations were conducted using three distinct body composition indices. The measurement was not performed due to potential influencing factors, including tendon thickening or contracture, which could compromise the integrity of the hand grip test results.

This study was conducted by the principles outlined in the Declaration of Helsinki. All participants provided written informed consent before their inclusion. The research protocol received approval from the Research Ethics Committee at the Dokuz Eylul University (2019-32-39).

### Statistical Analysis

The data were presented as percentages for categorical variables and median (interquartile range, IQR) for continuous variables. Group comparisons were made as appropriate using Student’s unpaired 2-tailed *t*-test or the Mann–Whitney test. The chi-square test was used to compare categorical variables. Spearman’s rank correlation coefficient was used to test for an association between numerical variables. Binary logistic regression analysis was conducted to identify the primary risk factors associated with sarcopenia in patients with SSc. A subsequent multivariate logistic regression analysis was performed, incorporating all variables identified as significant in the univariate analysis, to ascertain independent risk factors linked to sarcopenia. The relationship between sarcopenia (the dependent variable) and the independent variables—comprising SSc characteristics and laboratory test results—was quantified through the odds ratio (OR) along with the corresponding 95% confidence interval (CI). Results with a significance level of *p* < 0.05 were considered statistically significant.

## 3. Results

In this study involving 80 patients with systemic sclerosis, 91.3% were female, with a median age of 56.5 years (IQR 45–65). Approximately 30% of the participants were active smokers. Most patients presented with limited SSc, accounting for 70% of the cohort, and 71.3% had at least one comorbid condition. The median duration from symptom onset to diagnosis was 90 months (IQR 47.25–151). The median modified Rodnan skin score was 4 (1.25–10), while the Medsger score was 6 (4–7). Based on the International Physical Activity Questionnaire, only 12.5% of patients were considered active. During the past year, 27.5% (*n* = 22) reported experiencing falls, though none sustained fractures. According to the SARC-F questionnaire, 20% of patients were identified as being at risk for sarcopenia. Sarcopenia prevalence among patients demonstrated significant variability, with 5% (95% CI 0.1–9) identified through the ASMI, 8.8% (95% CI 2.4–15) via the FFMI, and a concerning 20% (95% CI 11–29) according to the SSMI. A summary of the patients’ demographic and clinical characteristics and sarcopenia assessments can be found in Table 1.

The median age of SSc patients with SARC-F scores ≥ 4 was 65 (range: 61–71.3) years, significantly higher than those with lower scores (*p* = 0.001). Additionally, these patients had a notably greater prevalence of at least one comorbidity (*p* = 0.001). Those with SARC-F scores of 4 or above demonstrated lower levels of physical activity and had longer times in the chair-stand test and 4 m gait speed assessments than SARC-F < 4 (*p* = 0.002, *p* = 0.025, *p* = 0.045, respectively). However, no statistically significant difference was found between the two groups regarding ASMI, FFMI, and SSMI. The clinical characteristics and sarcopenia assessments of patients with SARC-F scores ≥ 4 and those with lower scores are compared in Table 2.

Patients over 60 years of age demonstrated a lower level of physical activity, poorer chair-stand test outcome, and slower 4 m gait speeds (*p* = 0.013, *p* = 0.008, *p* = 0.001, respectively). Moreover, this age group reported a higher incidence of falls in the past year (*p* = 0.039). Furthermore, it was observed that at least one comorbidity was significantly more prevalent among these patients (*p* = 0.006). Of older patients, 27.5% (*n* = 11) had sarcopenia, according to SSMI. However, a statistically significant difference was not observed between the two groups concerning FFMI, ASMI, and SSMI. Table 3 compares the clinical characteristics and assessments of sarcopenia in patients over and under 60 years of age.

Our analysis indicated no significant differences in body composition measurements, such as SSMI, FFMI, and ASMI scores, between physically active and inactive patients. No significant correlation was found between these indices and SSc subtypes, mRSS, Medsger score, and disease duration. Table 4 presents the correlations between measurements of sarcopenia and various clinical variables in SSc patients. The analysis revealed no significant correlation between comorbidity and sarcopenia indices based on body composition; however, a notable association was observed with the incidence of falls (*p* = 0.002).

A thorough binary analysis revealed a significant correlation between an SARC-F score of ≥4 and variables such as age, pulmonary arterial hypertension, albumin levels, and C-reactive protein in patients with SSc (Supplementary Table S1). Moreover, a multivariate logistic regression analysis identified age as the only factor significantly influencing the SARC-F score, with an odds ratio of 1.081 (95% confidence interval: 1.012–1.154, *p* = 0.020) (see Supplementary Table S2).

## 4. Discussion

This study revealed that (1) patients with SARC-F scores ≥ 4 were generally older, and 20% of all patients were at risk of sarcopenia, according to SARC-F; (2) patients ≥ 60 years or with SARC-F scores ≥ 4 demonstrated reduced physical activity levels, poorer performance on chair-stand tests, and slower 4 m gait speed; (3) no significant correlations were observed between SSc subtypes, clinical findings, disease activity, duration, and body composition indices.

Sarcopenia and frailty are concepts that are frequently discussed together, yet they represent distinct syndromes. Frailty addresses a broader range of physical, social, and psychological factors. In contrast, sarcopenia is specifically characterized by a reduction in skeletal muscle mass, strength, and function, often associated with aging or chronic diseases [20].

The prevalence of sarcopenia demonstrates variability influenced by factors such as ethnicity, measurement methodologies, and the specific populations studied. According to the Asian Working Group for Sarcopenia definition, the prevalence of sarcopenia was 22.8% among Thai SSc patients [21]. A study using BIA found that 22.5% of SSc patients have sarcopenia [2]. In Italian SSc patients, sarcopenia was detected in 42% based on the Relative Skeletal Mass Index (RSMI) and 55% using handgrip strength [6]. The prevalence of sarcopenia in our patient cohort demonstrated significant variability, as assessed by various metrics: 5% identified using ASMI, 8.8% assessed with FFMI, and a concerning 20% according to SSMI. To accurately address sarcopenia within this specific disease group, it is essential to establish a clear and validated cut-off point. However, it should be noted that cut-off points for muscle mass have a more significant impact on sarcopenia prevalence than functional measurements [22]. We used the SMMI (weight) cut-off thresholds determined by Bahat et al. [14] in the Turkish population (37.4% and 33.6% for men and women, respectively).

Chronic inflammation, oxidative stress, mitochondrial dysfunction, diminished regenerative capacity, myosteatosis, and a reduced number of type II fiber satellite cells collectively contribute to the pathophysiology of sarcopenia [5]. Therefore, patients with rheumatic diseases such as SSc, rheumatoid arthritis, psoriatic arthritis, and systemic lupus erythematosus may be more prone to sarcopenia due to the pro-inflammatory state, physical inactivity, and corticosteroid therapy [23]. Our study observed no significant relationship between physical inactivity, as measured by the IPAQ, and FFMI, SSMI, or ASMI. Data from two studies involving 191 SSc patients were analyzed to explore the relationship between sarcopenia and C-reactive protein (CRP) levels. The findings indicated a significant association, showing that patients with sarcopenia had higher CRP levels [7]. In our study, elevated acute-phase reactants revealed only a weak positive correlation with gait speed. A cross-sectional study showed that a lower body BMI at initial assessment and higher levels of CRP at enrollment were associated with sarcopenia [21]. In our cohort, no significant difference was observed regarding sarcopenia and physical performance between those with and without BMI ≤ 18.5.

In the NHANES III dataset, sarcopenia was detected in 10% of women over the age of 60 according to skeletal muscle mass index [24]. Additionally, a study that examined 180 patients with SSc found that approximately half of those diagnosed with sarcopenia were over 60 years old [21]. Among our older patient population, 27.5% were assessed to have sarcopenia, as determined by the SSMI. However, Siegert et al. [2] found no significant age difference between patients with sarcopenia and those without.

In our study, consistent with the literature, no significant difference was found in the prevalence of sarcopenia among SSc subgroups [7,21]. Marighela et al. [25] showed that the only independent factor linked to an elevated risk of sarcopenia was the longer duration of the disease. Conversely, another study revealed that patients with short-duration SSc exhibited a higher delta FFMI than those with long-duration SSc [26]. Our study found no correlation between disease duration and sarcopenia. Caimmi et al. [27] demonstrated that patients with sarcopenia exhibited a longer duration of disease and worse Medsger severity scores. Another study indicated that several factors, including disease duration, mRSS, esophageal involvement, erythrocyte sedimentation rate, and antinuclear antibody titer, exhibited negative correlations with the RSMI. Conversely, the diffusion capacity of the lungs for carbon monoxide demonstrated a positive correlation with RSMI [6]. Moreover, SSc patients with low FFMI demonstrated higher disease activity index, mRSS, disease severity index, and disease duration [28]. Notably, we did not establish a significant association between mRSS or Medsger severity scores and the measurements of sarcopenia.

The number of comorbidities measured by the Charlson comorbidity index did not differ between German SSc patients with and without sarcopenia [2]. Our patients with comorbidity had higher SARC-F scores and a greater incidence of falls compared to those without comorbidity. However, comorbidities were not examined in detail according to the Charlson comorbidity index.

Although sarcopenia does not significantly increase mortality risk in patients with SSc, its early identification is critical [29]. Sarcopenia can enhance the risk of falls and adversely affect both quality of life and functional capacity. Patients who are older, have long disease durations, experience malnutrition, or use corticosteroids should be evaluated with the SARC-F score. A score of 4 or higher requires further body composition analysis and functional assessments, potentially leading to nutritional support and an exercise program.

BIA is a cost-effective, easy-to-use, and reproducible methodology. Its results correlated with magnetic resonance imaging predictions under standardized conditions [30]. However, our measurements utilizing BIA demonstrated variability in the prevalence of sarcopenia when evaluated with different indices. It is essential to conduct a well-structured study that includes a larger cohort of patients with SSc compared to healthy controls. This research will be critical in identifying the most suitable index and establishing appropriate cut-off values. There is also a definite need for longitudinal studies that evaluate both the progression of sarcopenia and the effectiveness of treatment protocols in SSc patients.

## 5. Conclusions

Our research highlights the prevalence of sarcopenia in patients with SSc, emphasizing the necessity for established cut-off values specific to this population. Early identification of sarcopenia is vital, as it facilitates the implementation of preventive strategies aimed at reducing disability, preserving functional abilities, and lowering hospitalization risks. Developing standardized criteria will significantly enhance the management of sarcopenia within the context of SSc.

## Figures and Tables

**Table 1:** Demographics, clinical characteristics, and sarcopenia-related assessment of the SSc patients.

Variable	*n* (%)
Gender, female	73 (91.3)
Age, years *	56.5 (45–65)
Smoker	24 (30)
SSc subtype	
dcSSc	23 (28.8)
lcSSc	56 (70)
Sine SSc	1 (1.3)
Overlap	29 (36.3)
Antinuclear antibody positive	
Anti-centromere antibody	27 (33.8)
Anti Scl-70 antibody	32 (40)
Presence of comorbidity	57 (71.3)
Diagnosis duration, months *	90 (47.25–151)
Body mass index (kg/m^2^) *	26.5 (23.75–31.88)
modified Rodnan skin score *	4 (1.25–10)
Medsger disease severity scale *	6 (4–7)
Pulmonary arterial hypertension	5 (6.3)
Interstitial lung disease	27 (33.8)
International Physical Activity Questionnaire	
Inactive	33 (41.3)
Moderate	37 (46.3)
Active	10 (12.5)
Serum albumin (g/dL) *	4.24 (4.0–4.36)
Total protein (g/dL) *	7.23 (6.99–7.57)
C-reactive protein (mg/L) *	3.7 (1.5–6.4)
Erythrocyte sedimentation rate (mm/h) *	18 (10.25–35)
Creatinin kinase (U/L) *	74 (54–108)
Steroid use	47 (58.8)
Immunosuppressive treatment	40 (50)
SARC-F score ≥4	16 (20)
Chair stand test, seconds *	11.8 (9.8–14.7)
4 m gait speed test, seconds *	3.87 (3.4–4.6)
≥0.8 m/s	71 (88.8)
Fat-free muscle mass index (kg/m^2^)	
Female	17.8 (16.7–19.8)
Male	18.4 (16.8–21.2)
Skeletal muscle mass index (%)	
Female	37.2 (34.2–40.2)
Male	41.8 (37.6–43.5)

* median, IQR.

**Table 2:** Comparison of demographics, clinical characteristics, and sarcopenia measurements of patients with SARC-F score ≥ 4 and <4.

Variable	SARC-F < 4 (*n* = 64)	SARCF ≥ 4 (*n* = 16)	*p*
Gender, female	58 (90.6)	15 (93.8)	0.670
Age, years*	51 (43.3–62.8)	65 (61–71.3)	0.001
Smoker	20 (31.3)	4 (25)	0.625
SSc subtype			
dcSSc	20 (31.3)	3 (18.8)	0.363
lcSSc	43 (67.2)	13 (81.3)	
Overlap	20 (31.3)	9 (56.3)	0.090
Antinuclear antibody positive			
Anti-centromere antibody	20 (31.3)	7 (43.8)	0.459
Anti Scl-70 antibody	26 (40.6)	6 (37.5)	
Presence of comorbidity	41 (64.1)	16 (100)	0.001
Diagnosis duration, months *	89 (47.3–138)	106 (39.8–189)	0.470
Body mass index (kg/m^2^) *	26.3 (23.8–31.7)	29.7 (23.7–35.2)	0.500
modified Rodnan skin score *	5 (1.25–10)	4 (0.5–10)	0.979
Medsger disease severity scale *	5.5 (3.25–7)	7 (4.25–9.75)	0.124
Pulmonary arterial hypertension	2 (3.1)	3 (18.8)	0.149
Interstitial lung disease	24 (37.5)	3 (18.8)	0.123
International Physical Activity Questionnaire			
Inactive	20 (31.3)	13 (81.3)	0.002
Moderate	35 (54.7)	2 (12.5)	
Active	9 (14.1)	1 (6.3)	
Serum albumin (g/dL) *	4.26 (4.06–4.37)	4.13 (3.86–4.32)	0.096
Total protein (g/dL) *	7.2 (7.0–7.6)	7.3 (6.7–7.4)	0.868
C-reactive protein (mg/L) *	3.1 (1.4–6)	4 (2.5–9.3)	0.141
Erythrocyte sedimentation rate (mm/h) *	18 (8.5–31.3)	19.5 (12–44.8)	0.197
Creatinin kinase (U/L) *	74 (55–108)	68 (39.5–119.8)	0.893
Steroid use	37 (57.5)	10 (62.5)	0.740
Immunosuppressive treatment	33 (51.6)	7 (43.8)	0.589
Chair-stand test, seconds *	11.5 (9.7–13.5)	15.6 (12.6–20.7)	0.025
4 m gait speed test, seconds *	3.7 (3.3–4.4)	4.2 (3.9–6.2)	0.045
Experiencing falls within the past year	14 (21.9)	8 (50)	0.057
ASMI (kg/m^2^)	7.3 (6.8–8.2)	7.8 (6.7–8.5)	0.395
Fat-free muscle mass index (kg/m^2^)	17.6 (16.7–19.4)	18.8 (16.2–20.5)	0.525
Skeletal muscle mass index, %	37.6 (34.7–40.3)	36.8 (33.7–40.6)	0.688

Abbreviations: ASMI; appendicular skeletal muscle mass index; * median, IQR.

**Table 3:** Comparison of demographics, clinical characteristics, and sarcopenia measurements of patients with age ≥ 60 and <60 years.

**Variable**	**<60 Years, *n* = 40** ***n* (%)**	**≥60 Years, *n* = 40** ***n* (%)**	***p***
Gender, female	36 (90)	37 (92.5)	0.697
Age, years *	45 (38.5–50)	65 (61–69)	0.001
Smoker	15 (37.5)	9 (22.5)	0.147
SSc subtype			
dcSSc	12 (30)	11 (27.5)	1.000
lcSSc	27 (67.5)	29 (72.5)	
Antinuclear antibody positive			
Anti-centromere antibody	13 (32.5)	14 (35)	0.406
Anti Scl-70 antibody	15 (37.5)	17 (42.5)	
Presence of comorbidity	23 (57.5)	34 (85)	0.006
Diagnosis duration, months *	85 (33.5–138)	91.5 (48–166.7)	0.176
Body mass index (kg/m^2^) *	25.9 (23.6–28.8)	29.8 (24.1–33.9)	0.116
modified Rodnan skin score *	3 (0.25–10)	5 (2–10.7)	0.747
Medsger disease severity scale *	6 (3.2–7.7)	5.5 (4–7)	0.777
International Physical Activity Questionnaire			
Inactive	10 (25)	23 (57.5)	0.013
Moderate	24 (60)	13 (32.5)	
Active	6 (15)	4 (10)	
Serum albumin (g/dL) *	4.27 (4.05–4.37)	4.15 (3.96–4.36)	0.136
Total protein (g/dL) *	7.22 (7.0–7.63)	7.24 (6.93–7.39)	0.170
C-reactive protein (mg/L) *	2.55 (0.9–4.65)	4.4 (2.6–9.3)	0.015
Erythrocyte sedimentation rate (mm/h) *	15.5 (7.25–24.5)	20 (12.2–39.5)	0.054
Creatinin kinase (U/L) *	73.5 (56–103.2)	74 (52–120.5)	0.239
SARC-F score ≥4	2 (5)	14 (35)	0.001
Chair stand test, seconds *	10.9 (9–13)	13.5 (11.1–16.6)	0.008
4 m gait speed test, seconds *	3.5 (3.2–3.9)	4.3 (3.8–5)	0.001
Experiencing falls within the past year	7 (17.5)	15 (37.5)	0.039
ASMI (kg/m^2^) *	7.0 (6.7–7.6)	7.7 (6.8–8.6)	0.154
Fat-free muscle mass index (kg/m^2^) *	17.2 (16.6–18.7)	18.8 (16.8–20.4)	0.338
Skeletal muscle mass index (%) *	38.4 (36.1–40.7)	36.4 (33.2–39.9)	0.109

Abbreviations: ASMI; appendicular skeletal muscle mass index; * median, IQR.

**Table 4:** Correlations between sarcopenia measurements and clinical variables in systemic sclerosis patients.

	Chair-Stand Test (Seconds)	Gaid Speed (Seconds)	SARC-F	CRP (mg/L)	Albumin (g/dL)	SSMI (%)
Age (years)	0.429	0.617	0.407	0.351	−0.211	−0.316
	0.001	0.001	0.001	0.001	0.060	0.004
Chair-stand test (seconds)	1.000	0.541	0.502	0.156	−0.211	−0.281
		0.001	0.001	0.170	0.060	0.012
Gaid speed (seconds)	0.541	1.000	0.447	0.244	−0.222	−0.416
	0.001		0.001	0.030	0.047	0.001

Abbreviations: CRP; C-reactive protein; SSMI; skeletal muscle mass index. Spearman correlation.

## Data Availability

The data that support the findings of this study are available from the correspondence author upon a reasonable request.

## Supplementary Materials

The following supporting information can be downloaded at https://www.mdpi.com/article/10.3390/jcm14051573/s1: Table S1: Univariate analysis for SARC-F ≥ 4; Table S2: Multivariate analysis for SARC-F ≥ 4.
