# Supplementary material for: Assessment of Factors Related to Sarcopenia in Patients with Systemic Sclerosis
Source: J Clin Med. 2025 Feb 26;14(5):1573. doi: 10.3390/jcm14051573 (PMC11900986; doi:10.3390/jcm14051573)
Supplement: Supplementary file 1 [file jcm-14-01573-s001.zip › jcm-3483674-supplementary.pdf]

*Supplementary Material*

# Assessment of factors related to sarcopenia in patients with systemic sclerosis

**Table S1.** Univariate analysis for SARC-F ≥4.

| | B | p | OR | 95% C.I.for OR | |
|---|---|---|---|---|---|
| | | | | Lower | Upper |
| **Age** | **0.092** | **0.004** | 1.096 | 1.030 | 1.166 |
| **PAH** | 1.968 | **0.041** | 7.154 | 1.085 | 47.186 |
| **Albumin** | -2.165 | **0.034** | 0.115 | 0.016 | 0.846 |
| **CRP** | 0.086 | **0.033** | 1.090 | 1.007 | 1.180 |

Abbreviations: C.I. confidence interval, CRP; C reactive protein, OR; odds ratio, PAH: pulmonary arterial hypertension.

**Table S2.** Multivariate analysis for SARC-F ≥4.

| | B | p | OR | 95% C.I.for OR | |
|---|---|---|---|---|---|
| | | | | Lower | Upper |
| **Age** | **0.078** | **0.020** | 1.081 | 1.012 | 1.154 |
| **PAH** | 1.191 | 0.276 | 3.289 | 0.387 | 27.964 |
| **Albumin** | -1.324 | 0.328 | 0.266 | 0.019 | 3.774 |
| **CRP** | 0.036 | 0.408 | 1.037 | 0.952 | 1.130 |

Abbreviations: C.I. confidence interval, CRP; C reactive protein, OR; odds ratio, PAH: pulmonary arterial hypertension.
